# Mechanical Characterization of Two-Segment Free-Standing ZnO Nanowires Using Lateral Force Microscopy

**DOI:** 10.3390/nano12234120

**Published:** 2022-11-22

**Authors:** János Volk, János Radó, Zsófia Baji, Róbert Erdélyi

**Affiliations:** 1 Centre for Energy Research, Institute of Technical Physics and Materials Science, Konkoly-Thege M. út 29–33, 1121 Budapest, Hungary; 2 Faculty of Information Technology and Bionics, Pázmány Péter Catholic University, Práter utca. 50/A, 1083 Budapest, Hungary

**Keywords:** nanomechanical characterization, bending modulus, ZnO nanowire, atomic layer deposition, Euler–Bernoulli beam theory, piezotronics, finite element analysis, atomic force microscopy

## Abstract

Mechanical characterization of quasi one-dimensional nanostructures is essential for the design of novel nanoelectromechanical systems. However, the results obtained on basic mechanical quantities, such as Young’s modulus and fracture strength, show significant standard deviation in the literature. This is partly because of diversity in the quality of the nanowire, and partly because of inappropriately performed mechanical tests and simplified mechanical models. Here we present orientation-controlled bending and fracture studies on wet chemically grown vertical ZnO nanowires, using lateral force microscopy. The lateral force signal of the atomic force microscope was calibrated by a diamagnetic levitation spring system. By acquiring the bending curves of 14 nanowires, and applying a two-segment mechanical model, an average bending modulus of 108 ± 17 GPa was obtained, which was 23% lower than the Young’s modulus of bulk ZnO in the [0001] direction. It was also found that the average fracture strain and stress inside the nanowire was above 3.1 ± 0.3 % and 3.3 ± 0.3 GPa, respectively. However, the fracture of the nanowires was governed by the quality of the nanowire/substrate interface. The demonstrated technique is a relatively simple and productive way for the accurate mechanical characterization of vertical nanowire arrays.

## 1. Introduction

The mechanical properties of nanowires/nanotubes (NWs/NTs) are of high relevance to many emerging applications, from flexible and stretchable sensors [1], through to piezotronic devices [2], and nanoelectromechanical systems (NEMSs) [3]. For most bulk single crystals all the relevant mechanical quantities, even the individual tensor components, are well known, and have been for decades. However, mechanical characterization of nanosized objects is highly challenging, and it is restricted mainly to the most relevant mechanical quantities, such as Young’s modulus, fracture/tensile strength, and toughness. Moreover, these quantities often show strong size and surface effects. Therefore, the reported values for most of the metallic, ionic, and covalent NWs show large standard deviation, and a general consensus is still missing [4]. 

Apart from a few early reports [5,6], the research on the mechanical properties of elongated micro- and nano-structures intensified about 25 years ago [7]. Since then, several techniques have been developed [8], which can be classified by various aspects. The NWs/NTs can be axially stretched, bent, or brought into resonance by an electric field. During the mechanical characterization in a microscope, NWs/NTs can be clamped, either on-purpose to the testbed or naturally to the substrate.

The axial load can be performed by clamping the nanostructures in between two atomic force microscope (AFM) cantilever tips and subjecting them to tension or compression loads in a scanning electron microscope (SEM) [9,10]. The microelectromechanical system (MEMS) was used as the test platform for mechanical characterization of nanofibers under optical microscope [11,12], or transmission electron microscope (TEM) [13]. Yield strength of suspended NWs can also be directly characterized by AFM [14,15]. However, all the above-listed methods require tedious specimen preparation, and, therefore, a large statistics study is troublesome. Moreover, as was pointed out by Qin et al. in [16] and Murphy et al. in [17], the widely used electron beam assisted deposition (EBAD) technique does not provide sufficiently tight clamping, which questions the credibility of the literature values in this class. Therefore, mechanical characterization of as-grown vertical NWs is highly preferred. On the one hand, the substrate provides a stiff natural clamping for nanocrystals, and, on the other hand, it does not require a time-consuming NW transferring process. 

Young’s modulus of free-standing vertical NWs can be determined by electric field-induced resonant frequency analysis, where the resonance is induced by applying an alternating electric field between the conducting sample and a sharp metal probe [18]. The main disadvantages of the method are its dependency on the electric properties of the nanostructures and its inability to examine low aspect ratio rigid nanopillars. A scanning electron microscope (SEM) equipped with nanomanipulator arms can be used to carry out in-situ static bending tests, even on shorter NWs [19,20]. By evaluating the deflection and curvature, local strain can be evaluated. For stress and Young’s modulus measurements, the bending load has to be calibrated. In a previous work, we showed that this could be done by mounting a calibrated AFM cantilever at the end of a robotic arm [18]. However, it required a carefully performed cleavage of the sensitive NW covered sample to ensure sidewise perpendicular access for the AFM tip and a detailed video analysis for each NW bending test to measure NW and AFM tip deflections. 

In contrast to the listed techniques, direct lateral force microscopy (LFM) measurement of vertical NWs is free of all the addressed drawbacks, assuming that the probe is appropriately calibrated. It is fast, and does not require a vacuum environment or additional sample preparation after bottom-up growth of the nanocrystals. During AFM scanning of the NWs, both cantilever deflection and torsion are monitored by a position sensitive photo detector (PSPD). The obtained normal and lateral force signals can provide information on normal and lateral forces acting on the tip. For instance, well controlled bending can be performed by simultaneously acquiring the topography and lateral PSPD signal images of aligned vertical NWs in the AFM contact mode [21]. However, in the usually applied indirect calibration methods many error sources exist, and, therefore, the mechanical quantities obtained in the literature must be carefully handled. While the spring constant of the cantilever probe in the normal direction (and, hence, the normal force acting on the probe) can be fairly well calibrated by resonant frequency or thermal noise methods [22], determination of the torsional compliance is usually troublesome. Moreover, the lateral PSPD signal depends both on the optical alignment of the instrument and on the tip geometry, where even the normal load can contribute to the lateral force signal, usually referred to as crosstalk effect.

Nevertheless, in the last three decades, several methods were developed for lateral force calibration, such as optical geometry [23], static friction [24], vertical lever [25], wedge [26,27], test probe [28,29], and diamagnetic lateral force calibration (D-LFC) [30]. In the latter technique, first proposed by Li et al. [30], a sheet of pyrolytic graphite (PG) is levitated by a strong magnetic field as a reference spring to apply a known lateral force on the AFM probe. Due to the direct nature of the method, the applied loads and, hence, the elastic moduli can be determined with high confidence, without knowing the parameters (e.g., geometry, resonant frequency) of the cantilever. Although the technique is mainly dedicated to detection of frictional force at small scale, in this report we present its capability for measuring the bending modulus (Young’s modulus measured by bending; *E_BM_*) and fracture strength of individual vertical NWs. 

We chose ZnO NW array to introduce our LFM based nanomechanical characterization method. The reported Young’s modulus values for ZnO are very strongly scattered in the literature [8,31,32,33]. Moreover, the sign and physical origin of the size effect are controversial [4]. Beyond the instrumental challenges mentioned, the significant deviation can also be attributed to the simplified geometrical model used for the mechanical calculation. In all these reports, ZnO NWs were described by a right circular cylinder. However, bottom-up grown NWs are always tapered, i.e., the diameter decreases toward the tip of the NW. Moreover, the cross-section of the wurtzite ZnO is a hexagon with a side *a*, so, depending on the viewing direction compared to the crystallographic orientation of the NW, the visible diameter changes in the range of √3*a* to 2*a*. In this report, crystallographically-aligned ZnO NWs were epitaxially grown by means of the selective area wet chemical method, showing non-uniform cross section along their long symmetry axis (*c*-axis). Though the most accurate mechanical analysis could be conducted by finite element analysis (FEA), we proposed a two-segment analytical model, which could be better automatized to collect higher statistics. In this report, FEA was used only for verification of the analytical model. As far as the authors are aware, this is the first report on the mechanical characterization of nanostructures using such a calibrated LFM system. Compared to the above-listed nanomechanical characterization techniques, the proposed technique does not require the transfer and clamping of individual NWs, yet, nevertheless, it provides quantitative loading force information. The proposed technique can be adapted to other semiconductor and metallic NWs/NTs, providing a valuable tool for the design of next generation energy harvesters, ultrahigh frequency nanoresonators, nanoelectromechanical systems, high-strength composites and flexible electronics [4].The paper is organized as follows: Section 2 describes the applied methods in terms of growth, calibration, and bending; Section 3 summarizes the obtained results and provides an analysis; and, finally, conclusions are given in Section 4.

## 2. Materials and Methods

### 2.1. Growth of ZnO Nanowire Arrays

Vertical ZnO NWs were synthesized by means of the selective area wet epitaxial growth method [34]. As was pointed out earlier, an appropriate choice of seed layer and the epitaxial growth of ZnO NWs on an epitaxial or highly textured layer, yields aligned ZnO NWs [35]. In this experiment, the atomic layer deposition (ALD) technique was used to grow the epitaxial ZnO seed layer at 300 °C in 500 cycles onto a 6 µm-thick metal–organic chemical vapor deposition (MOCVD)-grown GaN template (TDI, Inc.) [36].

**Figure 1:**
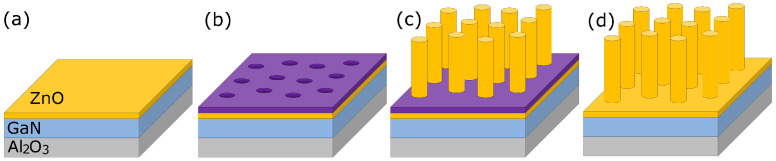
Schematic process flow of ZnO nanowire arrays. The processing steps were as follows: Deposition of epitaxial ZnO layer on GaN template using ALD (**a**), Pattern generation in PMMA by e-beam lithography (**b**), Wet epitaxial growth (**c**), and PMMA removal in acetone (**d**).

The steps of the process flow for the fabrication of ZnO NWs are illustrated in Figure 1. At first, the thoroughly cleaned (in acetone, ethanol, deionized water and by oxygen plasma) sample was spin-coated by a ~300 nm thick PMMA resist. An array of cylindrical holes, arranged in a triangular lattice (*Λ* = 500 nm), was generated in the resist using e-beam lithography in a Jeol IC 848-2 instrument (JEOL Ltd., Akishima, Tokyo, Japan) (Figure 1b). These holes served as nucleation windows in the PMMA layer for ZnO NW growth. The NWs were grown in aqueous solution, containing the same molar amount (4 mM) of zinc nitrate hexahydrate (Zn(NO_3_)_2_·6H_2_O) and hexamethylenetetramine ((CH_2_)_6_N_4_) during 3-hour-long nanostructure synthesis, at a set temperature of 85 °C (Figure 1c). After the slow cooling of the nutrient solution, the PMMA was removed in acetone, and the sample was thoroughly rinsed in deionized water (Figure 1d). The morphology of the NWs was examined by a LEO 1540XB SEM (Carl Zeiss AG, Oberkochen, Germany).

### 2.2. Lateral Force Calibration

The lateral force calibration method employed consisted of three steps: (i) characterization of the diamagnetic levitation spring; (ii) instrumental calibration to reveal the relationship between the photodetector signal and the applied lateral force; and (iii) determination of the lateral spring constant of the applied AFM probe, by pressing it against a rigid step. Steps (i) and (ii) were based on the technique proposed by Li et al. in [30] and are briefly described in the following paragraphs.

**Figure 2:**
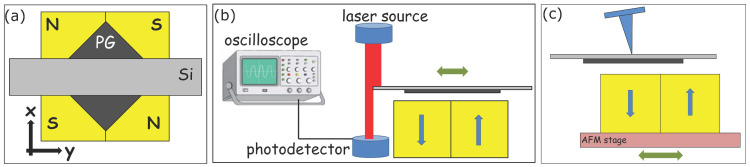
Schematic of D-LFC method: a pyrolytic graphite sheet-Si wafer assembly levitated by a strong magnetic field, drawn from top-view (**a**); laser displacement tracer setup to determine the angular frequency of the levitating system (**b**); diamagnetic levitation spring system mounted on the AFM stage to calibrate the lateral PSPD output (**c**) Blue arrows show the polarity of the magnets, green arrows indicate the direction of the movement of the sample and the AFM stage.

In our experiment, four neodymium-iron-boron (NdFeB) permanent magnet cubes (3 × 3 × 3 mm^3^) were used to levitate a square-shaped PG sheet (4 × 4 mm^2^). In order to avoid contaminating the AFM probe, and also to provide a chopper for the calibrating laser beam, as described below, a rectangular Si wafer with extended length (2 × 8 mm^2^) was glued on the top (Figure 2a). By placing the PG–Si assembly above the magnets, we obtained a standard spring-mass system which could vibrate harmonically in air. Since the vibration amplitude decayed slowly, by neglecting the damping the motion could be described by the differential equation of a simple harmonic oscillator. Hence, the magnetic spring constant could be calculated by:

(1) $$k_{PG}=m\omega^{2},$$

where *m* is the mass of the PG–Si assembly and *ω* is the angular frequency of the motion.

The angular frequency was experimentally determined in an optical manner, as depicted in Figure 2b. The laser beam of a simple laser pointer was partially blocked by the edge of the Si wafer. The PG sheet was pushed slightly off the center parallel with the long symmetry axis of the Si, and the transmitted light intensity was detected by a photodiode. The signal of the diode was monitored by a digital oscilloscope and the discrete Fourier transform (DFT) of the signal provided the frequency of the motion. Note that out of the two possible perpendicular orientations of the levitating PG–Si assembly (long symmetry axis of the Si wafer parallel with *x* or *y* direction in Figure 2a) only one was stable without beat frequency.

In order to ensure that the deflection of the NWs was significantly higher than the lateral torsion of the tip, instead of a common contact probe we chose a medium soft probe (BudgetSensors Multi75-G), having a nominal normal stiffness of 3 N/m and, hence, higher lateral stiffness compared to typical contact tips. For the calibration of the lateral forces acting on the AFM probe, the diamagnetic levitation system was mounted on the stage of an AIST-NT SmartSPM 1010 AFM (Figure 2c). The tip was engaged on the middle of the Si sheet, and the magnets, together with the AFM stage. were reciprocated by the scanner in the ±10 µm range, while the normal load was held fixed by the feedback loop. Since the spring constant of the PG–Si assembly and the lateral spring constant of the probe differed by orders of magnitude, the PG sheet, together with the tip, remained stationary, except for a slight cantilever twist within a few nanometers balancing the excursion of the magnets. For a linear system to convert the lateral (*V_lat_*) and normal (*V_norm_*) PSPD output to the lateral spring force acting on the probe (*F_lat_*) we needed two quantities:

(2) $$F_{lat}=\alpha_{ll}V_{lat}+\alpha_{ln}V_{norm}$$

where *α_ll_* and *α_ln_* are the force constants, while the displacement of the PG is neglected. The value *α_ln_* is usually referred to as crosstalk coefficient. By recording *V_lat_* against the lateral displacement of the magnet (*Y_stage_*) on AFM stage at different *V_norm_* signals (usually called set points) the force constants of the system could be approximated by two linear fittings:

(3) $$\alpha_{ll}=\frac{\partial F_{lat}}{\partial V_{lat}}\approx {{\left(\frac{\Delta V_{lat}}{k_{PG}\Delta Y_{stage}}\right)}^{-1}|}_{V_{norm}}$$

(4) $$\alpha_{ln}=\frac{\partial F_{lat}}{\partial V_{norm}}\approx {{\left(\frac{\Delta V_{norm}}{k_{PG}\Delta Y_{stage}}\right)}^{-1}|}_{V_{lat}}$$

Although, by means of the above-described procedure, the relationship between the PSPD signals and lateral force was obtained, it was also necessary to determine the lateral spring constant of the cantilever to distinguish the tip torsion from NW deflection during the NW bending test. Therefore, on the analogy of normal force calibration, we carried out a simple experiment to determine the lateral spring constant of the cantilever. A silicon sample, with a sharp rectangular step in parallel with the long symmetry axis of the cantilever, was scanned right and left along a line beneath the probe alongside deactivated feedback, and the distance between them was gradually decreased. When the very end of the tip of the sample was reached, indicated by the appearance of *V_lat_* and *V_norm_* signals, the *z*-approach was stopped, and the *V_lat_* and *V_norm_* vs. lateral stage displacement curves were recorded. Hence, the quotient of the lateral spring force acting on the probe, and the stage displacement during the torsion of the tip at the edge, corresponded to the lateral spring constant of the Si tip $\left(k_{lat}^{tip}\right)$.

### 2.3. Bending Test

The AFM stage with the NW array was scanned beneath the probe under deactivated feedback loop in a square area. During the approaching step, the distance between the perpendicularly standing NWs and the edge of the probe was gradually decreased until *V_lat_* and *V_norm_* signals were detected. The scan direction was perpendicular to the long symmetry axis of the cantilever. When the top of the rods was reached, as indicated by the appearance of both signals, the approach was stopped. Hence, the NWs were bent at their free end along the $\langle 1\bar{1}00\rangle$ lateral direction and we assumed, that the very end of the probe touched only the top region of the NWs. *V_lat_* and *V_norm_* images in constant height mode, i.e., without feedback, were recorded, in order to evaluate *E_BM_* and fracture strain.

## 3. Results and Discussion

### 3.1. ZnO Nanowire Arrays

**Figure 3:**
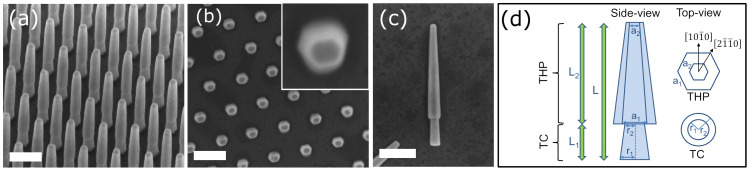
Tilted (**a**) and top-view (**b**) scanning electron micrographs of vertical ZnO nanowires grown on an atomic layer deposited ZnO on GaN/c-Al_2_O_3_ (0001). Hexagonal top and lower facets are shown in the magnified image (**b** inset). Side view image taken on a lying nanowire, illustrating the non-uniform cross section along the vertical axis (**c**). The length of the white scale bars was 500 nm. The geometry of the nanowires was divided into a lower truncated cone (TC) and an upper truncated hexagonal pyramid (THP) during the evaluation of the bending experiment (**d**).

The SEM study revealed that the c-axis oriented NWs were standing perpendicularly on the substrate (Figure 3a) and the hexagonal cross sections were collectively aligned (Figure 3b), which proved the single crystal character of the ALD ZnO seed layer. According to image analysis, the average length and diameter at the bottom were 1514 ± 32 nm and 165 ± 7 nm, respectively. The NWs were built up from two segments (Figure 3c–d): a ~320 nm high bottom part, having the shape of a truncated cone (TC), which was formed by filling the hole in the PMMA layer, and a longer upper part, having the shape of a truncated hexagonal pyramid (THP), resulting from free chemical growth above the PMMA [37]. Since the length homogeneity of the NWs was considerably high, several NWs could be bent by the AFM probe at their top, while scanning a single square area at an appropriately adjusted height.

### 3.2. Lateral Force Calibration

**Figure 4:**
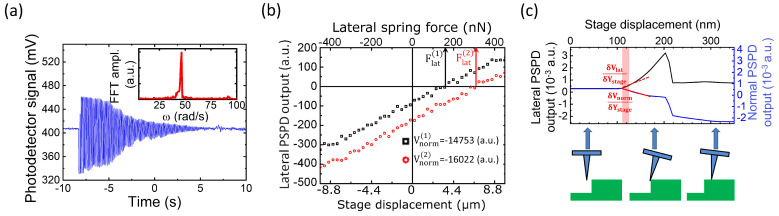
Three-step calibration procedure. At first, the free vibration of the magnetically levitated spring was recorded (**a**) to determine the resonant angular frequency by FFT (a inset). The lateral force acting on the probe was calibrated by the magnetic spring (**b**). Finally, the lateral (torsional) spring constant of the cantilever was evaluated by scanning a rigid rectangular step (**c**).

The optical monitoring of the diamagnetic levitation system revealed the in-plane direction (*y*) in which the vibration could be described with a single eigenfrequency (Figure 4a). From the obtained angular resonant frequency (*ω* = 46.49 rad/s), and from the measured mass (*m* = 21.0 mg), a lateral spring constant of *k_PG_* = 0.045 N/m was calculated for the magnetically levitated PG sheet.

The result of the lateral force calibration of the cantilever-AFM configuration is shown in Figure 4b. In accordance with expectations, the *V_lat_* signal was linear and reversible in the applied −10–10 μm lateral movement and in the corresponding −450–450 nN lateral force ranges. By changing the *V_norm_* setpoint, another parallelly shifted line was obtained (red circles in Figure 4b). The inverse slope of the data provided the lateral force constant of *α_ll_* (Equation (3)). On the other hand, according to Equation (4), *α_ln_* could be calculated using two parallel lines of different *V_norm_* values as follows:

(5) $$\alpha_{ln}=\frac{F_{lat}^{\left(2\right)}-F_{lat}^{\left(1\right)}}{V_{norm}^{\left(2\right)}-V_{norm}^{\left(1\right)}}$$

where $F_{lat}^{\left(1\right)}$, $F_{lat}^{\left(2\right)}$, $V_{norm}^{\left(1\right)}$ and $V_{norm}^{\left(2\right)}$ are depicted in Figure 4b. The average crosstalk coefficients for four sets of measurements were *α_ll_* = 1.83 ± 0.03 nN/a.u and *α_ln_* = −0.124 ± 0.04 nN/a.u. Note that the used instrument did not provide direct PSPD voltage value, but linearly proportional *V_lat_* and *V_norm_* signals in arbitrary units were recorded.

At given *α_ll_* and *α_ln_* instrumental calibration values, the lateral spring constant of the AFM tip was deduced from the scan taken on the Si step (Figure 4c). It consisted of three distinctive sections: (i) constant *V_lat_* and *V_norm_* off-set signals when the tip approached the step in contactless state; (ii) an ascending part (in absolute value) corresponding to the continuously increasing tip torsion at the Si step; and (iii) constant *V_lat_* and *V_norm_* signals on the right indicating a constant velocity tip movement on the top of the Si step, accompanied by finite kinetic friction. The ascendant section was non-linear, and, hence, the calculation of the lateral spring constant was not obvious. Since, during the NW bending test the lateral deflection of the AFM tip was in the range of 0–15 nm, we calculated the slope of the *V_lat_* and *V_norm_* curves in the low deflection range (pink region Figure 4c). Based on Equation (2) the lateral spring constant of the AFM was:

(6) $$k_{lat}^{tip}=\frac{\delta F_{lat}}{\delta Y_{stage}}=\alpha_{ll}\frac{\delta V_{lat}}{\delta Y_{stage}}+\alpha_{ln}\frac{\delta V_{norm}}{\delta Y_{stage}},$$

which was found to be $k_{lat}^{tip}$ = 28.7 N/m, though, due to the non-linearity mentioned, this value had significant uncertainty and might be underestimated compared to the higher slope regions. Nevertheless, as is shown later, during the bending analysis even this value was significantly higher than the spring constant of the NW. Therefore, it caused relatively low error when determining the NW deflection. On the other hand, $k_{lat}^{tip}$ was almost 3 orders of magnitude higher than that of the D-LFC spring (*k_PG_* = 0.045 N/m), which supported the assumption that the displacement of the tip and PG could be neglected when calculating the force constants using Equations (3) and (4).

### 3.3. Nanowire Scanning at Constant Tip Height

**Figure 5:**
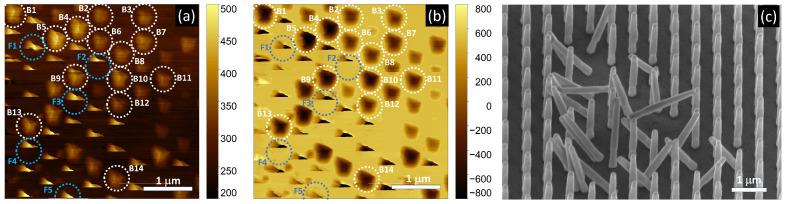
Lateral (**a**) and normal (**b**) PSPD signal maps taken upon the constant height scanning of the nanowires and tilt-view SEM image (**c**) taken on the same area after the scanning. Trapezoidal patterns in (**a**,**b**) show the sites where NWs remained unharmed (white circles), while triangles indicate fracture of the NWs (blue circles). Fourteen signal lines were selected to evaluate elastic NW bending (B1–B14) and five further ones to investigate the fracture (F1–F5).

*V_lat_* and *V_norm_* maps (Figure 5a,b, respectively) were recorded by scanning the NWs with the calibrated AFM probe in a horizontal window of 4 μm × 4 μm with subsequent directions of left-to-right and top-to-bottom. In contrast to conventional static and dynamic AFM operation modes, height was fixed at a carefully selected value to detect both bending and fracture events. At this height, the AFM probe was well above the substrate and flicked only the tip of the NWs. The obtained *V_lat_* and *V_norm_* maps showed irregular pentagon-like objects (white circles in Figure 5a,b) and triangle-like patterns (blue circles in Figure 5a,b) in a constant background, indicating the area where the tip did not interact with the NWs. The horizontal scanning in the maps, and, thus, the lateral loading force, corresponded to the $\langle 1\bar{1}00\rangle$ lateral direction. SEM observation revealed that the NWs in the pentagon sites showed no residual deflection (Figure 5c), indicating purely elastic bending. In contrast, missing NWs were found in the triangular sites, indicating fracture events (Figure 5c). In this subsection, the intact NWs, denoted by B1–B14 in Figure 5a,b, are studied while the fractured ones (F1–F5 in Figure 5a,b) are discussed later (Section 3.5).

**Figure 6:**
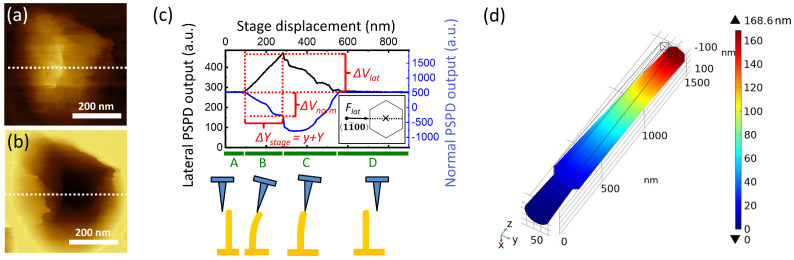
Typical bending curves (**c**) extracted from selected areas of *V_lat_* (**a**) and *V_norm_* (**b**) maps (B4 in Figure 5). White dashed lines in (**a**,**b**) show the selected lines corresponding to the maximal NW deflection. Calculated deformation for the same NW (B4) using Comsol FEA at maximal loading force right before the AFM tip-NW separation (**d**).

The irregular pentagons in *V_lat_* and *V_norm_* were the results of three factors: shape of the NW tip, shape of the AFM tip, and the compliance of the NW against the lateral point load. For quantitative mechanical analysis we extracted the line sections which showed the maximal bending deflection (white dashed lines in Figure 6a,b). Along these scanning lines the loading was assumed to be fully centered, and, hence, it caused no torsion or off-axis bending of the NWs (Figure 6c inset). Figure 6c shows typical bending curves and the schematic of the corresponding bending states in some characteristic points (A–D). The section started with constant values until the AFM contacted the NW (Figure 6c, A). The linearly changing region of *V_lat_* and *V_norm_* curves corresponded to the continuous bending of the NW, which resulted in a gradually increasing deflection and torsion of the cantilever (Figure 6c, B). The following irregular descent in the curve indicated slipping of the tip apex on the top facet of NW, accompanied by bending stress relaxation (Figure 6c, C). The increased fluctuation in the Vlat and Vnorm signals could be attributed to the surface roughness of the top facet of the NW. Finally, the AFM tip detached from the NW and signals returned to the original offset values (Figure 6c, D).

The projection of the linear region (Figure 6c, B) of the *V_lat_* curve on the horizontal axis, that is, the AFM stage movement (Δ*Y_stage_*), included both the maximal NW deflection (*y*) and lateral tip displacement (*Y*), whereas the projection on the corresponding vertical axis provided the maximal change of the lateral PSPD output during the bending (Δ*V_lat_*). Similarly, the projection of the bending section of the *V_norm_* curve on the corresponding vertical axis provided the absolute value of the maximal change of the normal PSPD output during the bending (Δ*V_norm_*). Therefore, the bending load at maximal deflection (*F_lat_*) could be deduced from Equation (2), using Δ*V_lat_* and Δ*V_norm_*. The maximal NW deflection could be determined by:

(7) $$y=\Delta Y_{stage}-\frac{F_{lat}}{k_{lat}^{tip}}$$

### 3.4. Calculation of the Bending Modulus

The bending modulus (*E_BM_*) of a vertical prismatic beam with uniform cross section affixed at the lower end and bent at the top by *F_lat_* could be calculated by solving the static Euler–Bernoulli equation:

(8) $$E_{BM}=\frac{F_{lat}L^{3}}{3yI},$$

where *L* is the vertical position of the applied load, which can be assumed to equal to the total length of the NW at the point of maximal deflection, and *I* is the second moment of inertia. The latter can be calculated either for hexagonal or circular cross section:

(9) $$I_{hex}=\frac{5\sqrt{3}}{16}a^{4}$$

(10) $$I_{circle}=\frac{\pi r^{4}}{4}$$

where *a* is the side of the hexagonal, and *r* is the radius of the circular cross section. Equation (10) is valid for both horizontal and vertical axes through the centroid. The fourth-power dependence of *a* and *r* in (9) and (10), respectively, could cause multiplied errors in a simplified model having uniform cross section along its length. Therefore, a non-uniform NW model was essential.

According to Castigliano’s theorem the total strain energy (*U*) of an elastic beam with respect to the load (*f_i_*) is equal to the deflection (*δ_i_*) corresponding to the load:

(11) $$\delta_{i}=\frac{\partial U}{\partial f_{i}}.$$

Assuming that the strain energy, due to traverse shear loading, is negligible, the strain energy resulting from bending of the NW could be calculated as follows:

(12) $$U=\int_{0}^{L}\frac{M^{2}}{2E_{BM}I\left(z\right)}dz,$$

where *z* runs along the *c*-axis of the NW from the bottom (*z* = 0) to *L*, and $M=F_{lat}\left(L-z\right)$ is the internal bending moment. Assuming that *E_BM_* is uniform in the NW, the deflection of NW could be constituted by the two separate segments of TC and THP:

(13) $$y=\frac{f}{E_{BM}}\int_{0}^{L}\frac{{\left(L-z\right)}^{2}}{I\left(z\right)}dz=\frac{f}{E_{BM}}\left[\int_{0}^{L_{1}}\frac{{\left(L-z\right)}^{2}}{I_{cir}\left(z\right)}dz+\int_{L_{1}}^{L}\frac{{\left(L-z\right)}^{2}}{I_{hex}\left(z\right)}dz\right].$$

Hence, using Equations (8) and (9), and calculating the integral, *E_BM_* could be expressed as:

(14) $$E_{BM}=\frac{f}{y}\left[\frac{4}{\pi }\frac{L_{1}\left(r_{1}^{2}L_{2}^{2}+r_{1}r_{2}LL_{2}+r_{2}^{2}L^{2}\right)}{3r_{1}^{3}r_{2}^{3}}+\frac{16}{5\sqrt{3}}\frac{L_{2}^{3}}{3a_{1}^{3}a_{2}}\right]$$

where *r*_1_/*r*_2_ is the radius of the lower/upper disk of TC; *a*_1_/*a*_2_ is the side of the lower/upper hexagon of THP; while *L*_1_ and *L*_2_ are their heights, respectively (Figure 3d). These geometrical parameters were determined from tilted and top view SEM images captured after the bending experiment from the scanned areas of the array, where some characteristic artifacts (e.g., contamination and nanowire vacancy) were used to identify the NWs to be evaluated (for example Figure 5c).

The analysis of B1–B14 NWs is summarized in Table 1. As is shown, the lateral bending force fell in the range of 233–488 nN, resulting in NW deflections of 115–183 nm. The average bending modulus was 108 ± 17 GPa, where ± indicated the standard deviation of the *E_BM_* values obtained. Table 1 also indicates the uncertainty of *E_BM_* for the individual NWs, which was calculated by the error propagation method using estimated errors for each parameter (listed in the header of Table 1). The uncertainty of *r*_1_, *r*_2_, *a*_1_, and *a*_2_ originated from the pixel size of the SEM image, whereas that of *L*_1_ and *L*_2_ were determined by the reading accuracy of TC/THC interface. The error of *F_lat_* was dictated by the uncertainty of the *α_nl_* and *α_nn_* parameters. In the whole procedure described so far, the least precisely defined quantity was $k_{lat}^{tip}$ because of the mentioned non-linearity in Figure 4c. However, it had a relatively low impact on *y* through Equation (6), since the deflection of the cantilever was roughly one order of magnitude lower than the deflection of the nanowire. Therefore, *δy* ≈ *δY_stage_*, where *δY_stage_* could be approximated by the step size during AFM scanning (4 nm).

Finite Element Analysis (FEA) was used to validate Equation (14). The simulation was performed by Comsol Multiphysics using the NW geometry, loading force and *E_BM_* value of B4 line in Table 1, beyond the default ZnO stiffness tensor and Poisson ratio of the software. The simulated maximal deflection (168 nm) agreed well with the measured value of 166 nm (Figure 6d).

The *E_BM_* obtained was 23% lower than the Young’s modulus of bulk ZnO in the [0001] direction (140 GPa) [37]. However, it was significantly higher than the value measured by Song et al. (29 GPa) in Ref. [21], who also applied an LFM technique on c-axis oriented vertical ZnO NWs. This discrepancy could be explained by the fact that they calculated the lateral spring constant assuming ideal geometry $k_{lat}=W^{2}k_{norm}/T^{2},$ where *k_norm_* was the normal spring constant and *W* and *T* were the width and thickness of the cantilever determined from SEM images. They determined the lateral sensitivity afterwards from the curve of lateral force vs. distance when the tip was scanned in contact mode on a flat surface. However, the thickness of the cantilever could be poorly measured by SEM, and, moreover, the lateral force calibration method they chose was indirect and neglected the crosstalk component, so, therefore, the obtained *E_BM_* became considerably uncertain. 

Further EBM values reported in the literature scatter in the range of 100–170 GPa for ZnO nanowires having similar diameter (100–200 nm) [4,8]. Both the resonance method and the lateral bending of double-side clamped NWs showed a larger NW-to-NW variation in EBM compared to our technique. This could either be attributed to the more uniform NWs or to the more accurate measurement technique described in this report. Moreover, none of the previous reports took into account the tapered geometry of the NW and the crystallographic direction of the bending direction, which could lead to significant inaccuracies in analysis.

### 3.5. Fracture Test of ZnO NWs

**Figure 7:**
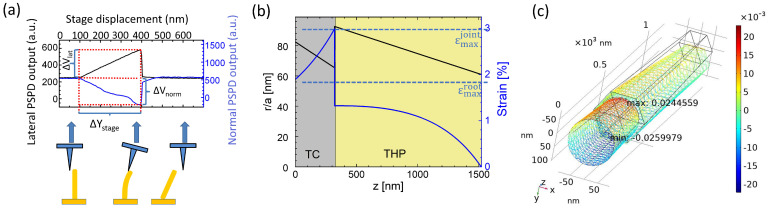
Bending curves of NW F2 taken in the scanning line of the fracture event (**a**). Calculated maximal strain at the surface from bottom to the top of the NW along the *z*-axis at changing radius (**b**). Comsol simulation of volumetric strain (**c**). The maximal value was located near to the interface of TC–THP segments.

As mentioned above, several fractures occurred upon scanning the NWs, partly because of non-zero scattering in NW heights and partly because of a slight incline of the sample surface, compared to the plane of the scanning. The fracture events could be identified by the triangular shaped objects in the *V_lat_* and *V_norm_* maps (black dashed circles in Figure 5a,b, respectively) in contrast to the pentagons, which were characteristic for intact NWs. Upon the left-to-right and then up-to-down probe scanning the sacrificed NWs showed, at first, similar bending lines as the intact ones. However, at a critical NW bending, *V_lat_* and *V_norm_* signals dropped to the zero-force value (i.e., the level of relaxed cantilever), indicating the fracture. The maximal lateral load right before the fracture could be calculated from the last scanning line in the same way as in Section 3.3., using the projection of the bending section of the *V_lat_* and *V_norm_* curves (Δ*V_lat_* and Δ*V_norm_*) (Figure 7a). The obtained maximal bending loads and deflections for five examined fractures (F1–F5) ranged from 561 nN to 703 nN and from 200 nm to 277 nm, respectively (Table 2). Since, after the fracture NW F1–F5 were not aligned anymore, the exact geometrical parameters could not be determined individually from the SEM image (Figure 5c). Instead, in this section, we used the average geometrical parameters shown in the last row of Table 1. Likewise, we used the average bending modulus when calculating the fracture strain.

Fracture strain could be estimated by calculating the maximal strain right before the breaking point. For NWs having uniform diameter the maximal strain was located on the surface of the NW at its root and could be calculated by the following equation:

(15) $$\varepsilon_{max}^{root}=\frac{3r_{1}}{L^{2}}y.$$

However, for two-segment NWs with tapered shafts the maximal strain could be located elsewhere, depending on the geometrical parameters. In this case, the strain at the outer surface of the NW along the *z*-axis was:

(16) $$\varepsilon_{max}=\frac{r\left(z\right)}{R\left(z\right)}=\frac{r\left(z\right)F_{lat}\left(L-z\right)}{E_{BM}I\left(z\right)}$$

where *r*(*z*) is the distance of the surface from the neutral centroid, i.e., it varied from *r*_1_ to *r*_2_ in TC and $\sqrt{3}/2a_{1}$ to $\sqrt{3}/2a_{2}$ in THP; and *R* is the radius of the local curvature. Indeed, using averaged geometrical parameters, the maximal strain was found to be right below the TC/THP joint (Figure 7b). It was also confirmed by FEA that the maximal volumetric strain and maximal stress were located at *z* = *L*_1_ (Figure 7c). However, as can be seen in the SEM image in Figure 5c, all the NWs broke at their roots (Figure 5c), i.e., the bottom plane of the TC was released from the seed layer. This effect could be attributed to the increased number of defects at the seed layer/NW interface, compared to the smooth transition zone of the TC/THP joint.

Table 2 lists the maximal strain and stress values (*σ* = *εE_BM_*) for both interfaces for each fractured NW. It could be concluded that the fracture strain and stress at the bottom were in the range of $\varepsilon_{max}^{root}=1.94\pm 0.19\%\;$ and $\sigma_{max}^{root}=2.09\pm 0.21\;\mathrm{GPa}$. However, in the NW itself it was above $\varepsilon_{max}^{joint}=3.06\pm 0.31\%\;$ and $\sigma_{max}^{joint}=3.30\pm 0.33\;\mathrm{GPa}.$ Both fracture stresses were significantly higher than the bulk value (0.1 GPa) [38], which could be attributed to the lower density of preexisting defects as the primary source of crack formation [39]. On the other hand, our *σ*_bot_ bending fracture strength was lower than the quantity (7.5 GPa) reported by Hoffmann et al. [19] on thermally evaporated nanowires having similar volumetric size (~0.025 μm^−3^). This suggested that the growth method had a decisive role on the quality of the substrate/nanostructure interface. A possible explanation of the modest fracture strength was either the lower quality of the hydrothermally-grown crystals (in the initial nucleation stage) or a residual PMMA which could remain in small quantities in the growth windows after developing the e-beam pattern and might hinder the full coverage of the selected growth area. The latter effect could be avoided by an alternative process, wherein the growth patterns were realized by focused ion beam [40] or ion milling [34]. The obtained lower limit of fracture strain inside the NW was close to the elastic strain limit of ~4% reported for similar NWs in a previous SEM micromanipulator-based experiment [20].

## 4. Conclusions

In summary, we demonstrated an accurate method for measuring the bending modulus and fracture strength of naturally clamped free-standing nanowires using lateral force microscopy. It requires a precisely calibrated AFM system and a sophisticated mechanical model which describes the tapered, two-segment character of the ZnO NW. The lateral force was calibrated with a soft diamagnetic levitation spring system. An Euler–Bernoulli beam theory-based analytical mechanical model was used to describe the segmented and tapered geometry of the NW. Due to the direct nature of the method, the elastic properties of highly ordered ZnO nanowires were measured with high confidence. The obtained average bending modulus of 108 ± 17 GPa was 23% lower than the Young’s modulus of bulk ZnO in the [0001] direction. Using the same technique, the average fracture strain and stress were also determined (1.94 ± 0.19% and 2.09 ± 0.21 GPa, respectively). The presented technique is a relatively simple, productive, and automatable method to determine the mechanical properties of various bottom-up grown nanowires. In addition, by collecting lateral force and electrical signals in parallel, using a conductive AFM tip, coupled electromechanical properties of ferroelectric and piezoelectric NWs could also be investigated.

## Figures and Tables

**Table 1:** Detailed parameters and results of the bending experiment on 14 NWs. The columns from left to the right denote: nanowire number (NW), lower (*r*_1_) and upper (*r*_2_) diameter of the truncated cone, lower (*a*_1_) and upper (*a*_2_) projected width of the truncated hexagonal pyramid, height of the truncated cone (*L*_1_), whole length of the nanowire (*L*), bending load (*F_lat_*), cantilever deflection (*Y*), nanowire deflection (*y*) and calculated bending modulus (*E_BM_*) using Equation (14).

NW [#] STD	*r*_1_ [nm] ±3	*r*_2_ [nm] ±3	*a*_1_ [nm] ±3	*a*_2_ [nm] ±3	*L*_1_ [nm] ±20	*L*_2_ [nm] ±20	*L* [nm]	*F_lat_* [nN] ±10	*Y* [nm] ±3	*y* [nm] ±5	*E_BM_* [GPa]
B1	80	69	90	58	326	1200	1526	343	12.0	146	120 ± 12
B2	82	63	98	62	318	1230	1548	249	8.7	130	93 ± 11
B3	79	62	94	65	326	1228	1554	238	8.3	123	107 ± 12
B4	87	71	103	64	341	1207	1548	445	15.5	166	100 ± 10
B5	83	69	88	62	304	1199	1503	488	17.0	183	124 ± 12
B6	82	67	88	60	326	1155	1481	233	8.1	142	78 ± 9
B7	82	65	104	62	303	1213	1516	270	9.4	137	82 ± 9
B8	85	63	96	60	311	1207	1518	238	8.3	115	94 ± 11
B9	87	67	90	62	333	1185	1518	416	14.5	166	113 ± 11
B10	78	66	92	59	313	1203	1516	346	12.0	134	132 ± 14
B11	79	62	91	58	303	1235	1538	300	10.5	136	129 ± 14
B12	87	68	84	58	307	1173	1480	283	9.8	133	102 ± 11
B13	87	65	88	65	324	1112	1436	392	13.6	144	105 ± 11
B14	79	64	91	60	324	1181	1505	345	12.0	138	129 ± 14
Average	82	65	93	61	319	1195	1514	327	11.4	142	108 + 17

**Table 2:** Results of the fracture tests on 5 NWs (F1–F5): applied load (*F_lat_*), NW deflection (*y*), calculated maximal strain/stress at the root of the NW ($\varepsilon_{max}^{root}/\sigma_{max}^{root}$ at *z* = 0) and at the joint of TC/THP segments ($\varepsilon_{max}^{joint}/\sigma_{max}^{joint}$ at *z* = *L*_1_). For the calculation average geometrical parameters and *E_BM_* were used (last raw in Table 1).

NW [#]	*F_lat_* [nN]	*y* [nN]	$\varepsilon_{max}^{root}$ [%]	$\sigma_{max}^{root}$ [GPa]	$\varepsilon_{max}^{joint}$ [%]	$\sigma_{max}^{joint}$ [GPa]
F1	569	242	1.82	1.96	2.86	3.09
F2	570	211	1.82	1.96	2.87	3.10
F3	635	221	2.02	2.19	3.19	3.45
F4	561	200	1.79	1.93	2.82	3.05
F5	703	277	2.24	2.42	3.53	3.82
Average	608 ± 61	230 ± 30	1.94 ± 0.19	2.09 ± 0.21	3.06 ± 0.31	3.30 ± 0.33

## Data Availability

The data presented in this study are available on reasonable request from the corresponding author.
